# Integration of liver gene co-expression networks and eGWAs analyses highlighted candidate regulators implicated in lipid metabolism in pigs

**DOI:** 10.1038/srep46539

**Published:** 2017-04-19

**Authors:** Maria Ballester, Yuliaxis Ramayo-Caldas, Manuel Revilla, Jordi Corominas, Anna Castelló, Jordi Estellé, Ana I. Fernández, Josep M. Folch

**Affiliations:** 1 Departament de Ciència Animal i dels Aliments, Facultat de Veterinària, Campus UAB, Bellaterra, 08193, Barcelona, Spain; 2 Plant and Animal Genomics, Centre de Recerca en Agrigenòmica (Consorci CSIC-IRTA-UAB-UB), Edifici CRAG, Campus UAB, Bellaterra, 08193, Barcelona, Spain; 3 IRTA, Genètica i Millora Animal, Torre Marimon, 08140 Caldes de Montbui, Spain; 4 Génétique Animale et Biologie Intégrative, Institut National de la Recherche Agronomique, AgroParisTech, Université Paris-Saclay, 78350, Jouy-en-Josas, France; 5 Departamento de Mejora Genética Animal, INIA, Ctra. de la Coruña km. 7, 28040, Madrid, Spain

## Abstract

In the present study, liver co-expression networks and expression Genome Wide Association Study (eGWAS) were performed to identify DNA variants and molecular pathways implicated in the functional regulatory mechanisms of meat quality traits in pigs. With this purpose, the liver mRNA expression of 44 candidates genes related with lipid metabolism was analysed in 111 Iberian x Landrace backcross animals. The eGWAS identified 92 eSNPs located in seven chromosomal regions and associated with eight genes: *CROT, CYP2U1, DGAT1, EGF, FABP1, FABP5, PLA2G12A*, and *PPARA*. Remarkably, *cis*-eSNPs associated with *FABP1* gene expression which may be determining the C18:2(n-6)/C18:3(n-3) ratio in backfat through the multiple interaction of DNA variants and genes were identified. Furthermore, a hotspot on SSC8 associated with the gene expression of eight genes was identified and the *TBCK* gene was pointed out as candidate gene regulating it. Our results also suggested that the PI3K-Akt-mTOR pathway plays an important role in the control of the analysed genes highlighting nuclear receptors as the NR3C1 or PPARA. Finally, sex-dimorphism associated with hepatic lipid metabolism was identified with over-representation of female-biased genes. These results increase our knowledge of the genetic architecture underlying fat composition traits.

Over the last decades, intra-muscular fat content and fatty acid composition have emerged as economically important traits due to their profound effect in meat quality^1^. Furthermore, with the large increase in obesity and other related metabolic diseases such as diabetes, fatty liver disease or atherosclerosis, meat fatty acid profile is also becoming a critical factor for consumers’ health^2^,^3^.

Fatty acid composition in pigs depends of physiological status, environmental factors such as nutrition^1^,^4^ and genetic factors^5^,^6^. In fact, whole genome scanning has revealed the presence of more than 2,200 quantitative trait *loci* (QTLs) for fatness and more than 670 QTLs for fat composition in the pig genome^7^. However, traditional approaches based on the classical single-gene-single-trait analysis had limited success in identifying causal mutations most probably due to the tiny proportion of phenotypic variation explained by the identified genetic variants in GWAS studies^8^,^9^. Furthermore, recent GWAS for backfat (BF) and intramuscular fatty acid (IMFA) composition have suggested pleiotropic effects of genetic variants for fatty acid composition traits^10^,^11^,^12^,^13^,^14^, indicating the complex genetic basis of lipid metabolism and, in consequence, fatty acid composition.

The development of genomic technologies has allowed the use of new approaches integrating genotypic and intermediate phenotypes such as mRNA expression data to detect DNA variants associated with gene expression levels that also associate with complex traits^15^,^16^,^17^. This approximation has succeed in identifying molecular gene networks and evidenced how variations in these networks better explain the functional mechanisms implicated in complex traits^15^,^16^. These new approaches together with gene co-expression network analyses have arisen as important tools to decipher the molecular mechanisms underlying complex biological process^18^,^19^.

Lipid metabolism involves genetic networks or molecular pathways that are interconnected and cross-regulated by hormones (peptides or lipids), transcription factors, cofactors and nuclear receptors^20^,^21^ that are tissue-specific and expressed at a relevant time in response to specific stimuli. In mammals, liver plays a key role in carbohydrate, amino acid and lipid metabolism, regulating whole-body energetic homeostasis. In pigs, liver is the principal organ implicated in the synthesis and secretion of very low density lipoproteins, *de novo* cholesterol synthesis and fatty acid oxidation, and together with the adipose tissue, but in a lesser extent, in *de novo* fatty acid synthesis^20^,^22^.

Recent studies in our group have identified differentially-expressed genes related with lipid metabolism in the liver transcriptome of two groups of pigs with phenotypically extreme IMFA composition in an Iberian x Landrace cross population^23^. Furthermore, in the same animal population, GWAS analyses have pointed out genomic regions significantly associated with IMFA composition^10^. In the present article, with the aim of further study the role of liver in determining meat quality traits in the Iberian x Landrace backcross population (BC1_LD), we have selected a group of 44 candidates genes related with lipid metabolism to study their expression pattern in liver. Therefore, we integrated genotypes and expression phenotypes to perform eGWAS analysis and co-expression networks that can help to increase our knowledge of fat content and fatty acid composition in pigs.

## Results and Discussion

### Selection of genes related to lipid metabolism in liver

The selection of genes used in the present study was based on previous results obtained by our group in which the study of the RNA-Seq liver transcriptome in two phenotypically extreme groups of animals for IMFA composition from an Iberian x Landrace cross revealed differentially-expressed genes playing an important role in lipid metabolism^23^. Thus, considering the multifunctional role that liver plays in lipid metabolism^20^, genes related with lipoprotein synthesis (*APOB*), cholesterol metabolism (*ABCG8, APOA2*, and *CYP7A1*), and induction of mitochondrial fatty acid oxidative metabolism (*LPIN1*) were selected. Furthermore, functional and positional candidate genes related with lipid metabolism and associated with the profile of IMFA composition in a GWAS study in the same animal cross^10^ were also selected (*ARNT, CYP2U1, EGF, ELOVL6, FABP5, HADH, HNF4A, MTTP, NFKB1, PLA2G12A, PLCB2*, and *USF1*, according pig genome assembly *Sscrofa10*.*2*). *ABCG8, APOA2, APOB*, and *CYP7A1* mapped also in the GWAS intervals associated with IMFA composition^10^. In order to complete the set of genes, we chose genes coding for proteins with different roles in liver lipid metabolism such as transporters (*FABP1* and *SCAP*), enzymes (*ACSM5, AGPAT2, CPT1A, CROT, DGAT1, ELOVL5, FADS1, FADS2, FADS3, LIPC, PEX2, PPAP2A*, and *SCD*) and transcriptional factors, cofactors or nuclear receptors (*ESRRA, FOXA1, HNF4G, KLF10, MLXIPL, NR1H3, NR2E1, POU2F1, PPARA, PPARD, PPARGC1A*, and *SREBF1*).

Next, liver mRNA expression of 48 genes (the 44 target lipid-related genes and 4 reference genes) was analised by Real Time-quantitative PCR (RT-qPCR) in 111 BC1_LD animals. Moderate to high mRNA expression levels in liver were observed for all genes with the exception of *NR2E1* that was expressed at low levels. Therefore, this gene was discarded for further analyses which were performed in 43 instead of 44 target genes. The *CYP7A1, FADS1, FADS2*, and *SCD1* genes presented the highest coefficients of variation (CV) (values ranging from 0.84 to 1.52) whereas the rest of genes presented values ranging from 0.18 (*USF1*) to 0.64 (*LPIN1*). Overall, the transcriptional factors, cofactors and nuclear receptors presented the lowest CV.

### Sex bias at liver co-expression gene network level

Taking into account that a pronounced sexual dimorphism has been described in the control of fatty acid homeostasis^24^,^25^, liver weighted gene co-expression networks separated by sex of the 43 candidate genes were built using PCIT algorithm^26^ to evaluate whether or not sexual dimorphism exists at the hepatic co-expression level in pigs. In fact, according to a differential expression analysis, 30 of the 43 genes already showed a sex biases (Supplementary Table S1).

**Figure 1:**
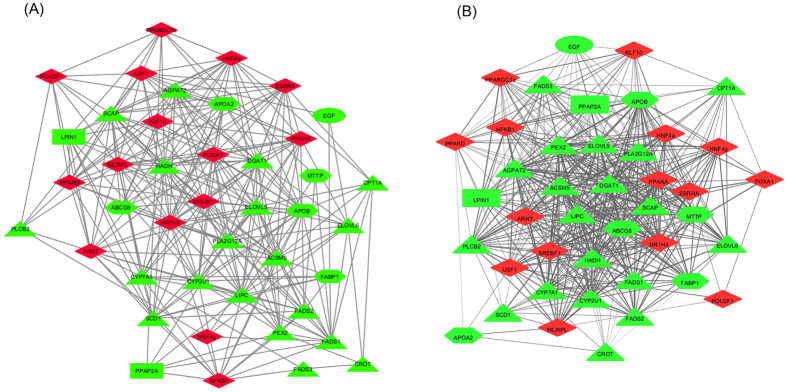
Co-expression network of the 43 selected genes using PCIT algorithm. (**A**) Female co-expression network; (**B**) Male co-expression network. Red color represents transcriptional regulators and green color represents enzymes and transporters. Node shape represents epidermal growth factor (ellipse), lipid-related enzymes (triangle), transfer and transport enzymes (hexagon), phosphatase enzymes (rectangle) and transcriptional regulators (diamond).

**Figure 2:**
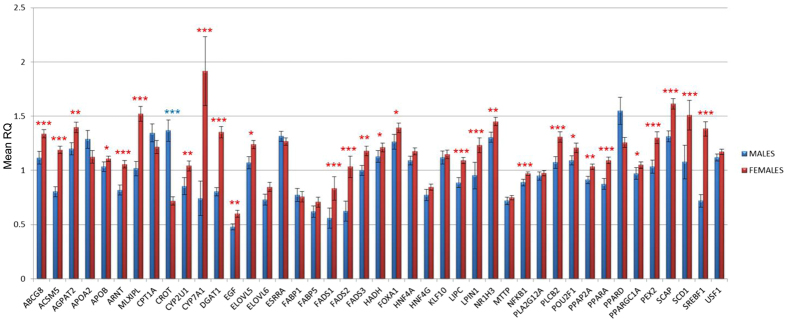
Comparison of liver gene expression levels of 43 lipid-related genes between males and females. Data represents means ± SEM. Significant differences between sexes are indicated as *<0.05, **<0.01, ***<0.001.

In both sexes, co-expression analyses revealed highly connected networks, which may be in accordance with the strong functional interconnections among the studied genes. Although topological parameters showed similar values between sexes (Supplementary Table S2), important differences were observed in the number of significant interactions between females (299 edges) and males (481 edges), indicating sexually dimorphic networks (Fig. 1). This result is in accordance with previous studies in which modules of highly differentially connected genes between males and females were detected in gene co-expression networks of adipose and liver mouse tissues^27^. In addition, based on network centralities, the relevance of individual genes differed according the sex in each network. For instance, considering the ten genes showing the highest centrality values, overlapping was observed only for six of them (*LIPC, PLA2G12A, ACSM5, ABCG8, PPARA*, and *CYP2U1*). In the male co-expression analysis the rest of genes showing the highest centrality values were *DGAT1, ELOLV5, PXMP3*, and *HADH*, whereas in the female co-expression network were *AGPAT2, ESRRA, HNF4A*, and *LXRA*. Interestingly, HNF4A is a key regulator of gene expression in liver^28^. Previous studies have determined the role of growth hormone-dependent transcription factors in the regulation of sex-specific gene expression in human, rat and mouse liver^25^,^29^. In consequence, this result joined with the bias observed in the female *PPARA* and *SREBP1* expression compared with males may explain the predominance of female biased expressed genes (Fig. 2) which is in agreement with the association of sex dimorphism in lipid metabolism described in other species^25^. This result highlights the relevance of including sex as co-factor in the model for eGWAS.

### Expression-based genome-wide association studies (eGWAS)

Taking into account that the activities of enzymes involved in lipid metabolism seem to be mainly regulated at the transcriptional level^24^, a GWAS was performed using gene expression levels of the previously 43 genes generated by RT-qPCR in 111 BC1_LD animals and the genotypes of 40,502 SNPs of the Porcine SNP60 BeadChip (Illumina).

Significant associations at whole-genome level were detected for eight of the 43 analysed genes: *CROT, CYP2U1, DGAT1, EGF, FABP1, FABP5, PLA2G12A*, and *PPARA*. A total of 92 significant eSNPs located in seven chromosomal regions on pig chromosomes SSC2, SSC3, SSC4, SSC8, and SSC13 were identified (Table 1). According to the Sscrofa10.2 assembly, of the 92 eSNPs, 54 (58.7%) were mapped in intergenic regions, 33 (35.9%) within genes whereas three and two SNPs were mapped in 5′ upstream and 3′ downstream gene regions, respectively. Most of the gene-associated SNPs were located in introns (81.8%) while only four were exonic and synonymous and one was a 3′ UTR variant (Supplementary Table S3). Considering *cis*-acting eSNPs as SNPs located within ±1 Megabase (Mb) of the transcription start or stop of the corresponding target gene, 15 eSNPs (16.3%) were classified as *cis*-acting eSNPs whereas the remaining 77 eSNPs (83.7%) have *trans* regulatory effects on the gene expression traits (Supplementary Table S3). These results agree with the fact that trans-acting regulation contributes notably to gene expression variation^30^, since genes seem to be regulated by several *trans*-acting regulators and only a few *cis*-acting regulators^31^.

### Functional network analysis of genes located in eQTLs

To go further in the characterization of the regulatory mechanisms that are influencing the gene expression phenotypes, gene annotation of the seven regulatory regions was performed. A total of 331 protein-coding genes, 13 miRNA, five pseudogenes, two rRNA, 19 snoRNA and 23 snRNA were annotated. From the 331 protein-coding genes with ensembl Gene ID, 171 were converted to human genes and submitted to IPA to perform a functional categorization (Supplementary Table S4). Remarkably, among the top-five associated networks, lipid metabolism was among the most represented functions (Supplementary Table S5) in which the PI3K complex is central in the network. PI3K activates Akt which in turn activates multiple downstream targets, one of the most important the mTOR pathway which plays a central role in hepatic lipid homeostasis^32^. Interestingly, the PI3K-Akt pathway has been also identified as a central in a muscle transcriptome study between individuals phenotypically extreme for IMFA composition^33^, and in a muscle eQTL analysis of 45 lipid-related genes^34^, suggesting an important role of this pathway in the genetic determination of fatty acid composition traits in the BC1_LD.

### *Cis*-eQTLs

**Figure 3:**
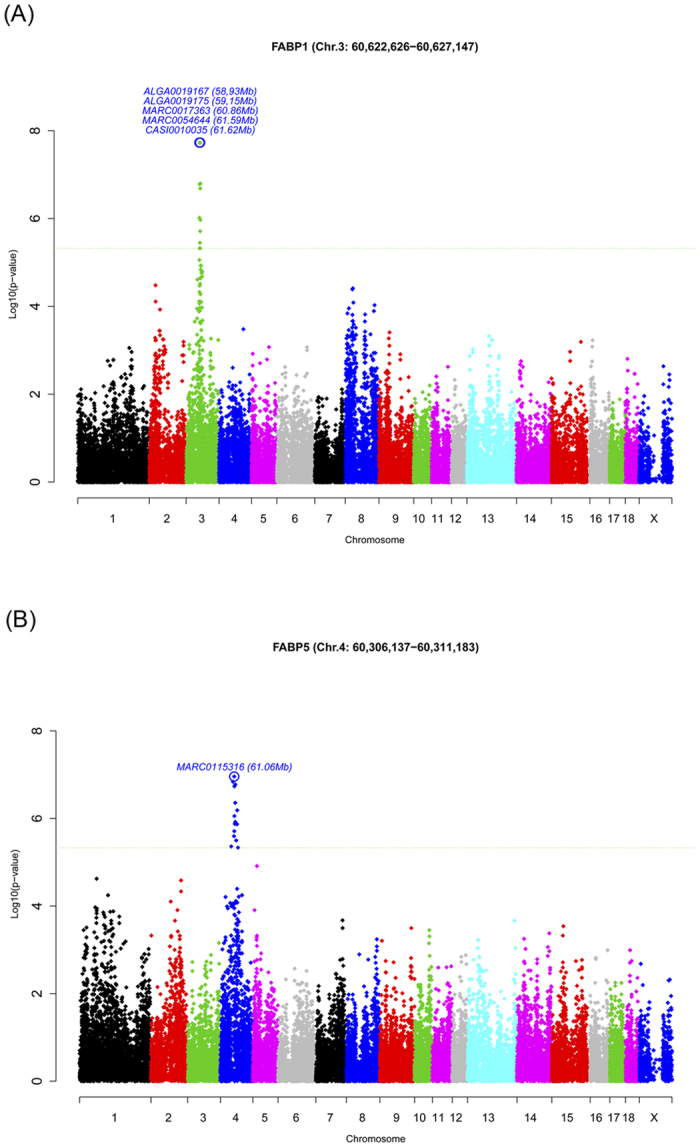
Plot of GWAS for *FABP1* and *FABP5* gene expression in liver. The X-axis represents chromosome positions in Mb relative to *Sscrofa10*.*2* assembly of the pig genome and the Y-axis shows the −log10 (p-value). Horizontal, dashed lines mark the genome-wide significance level (FDR-based q-value ≤ 0.01). Plot of eGWAS for (**A**) FABP1 gene expression in liver (**B**) FABP5 gene expression in liver.

The *FABP1* and *FABP5* genes, whose expression appeared to be associated with *cis*-eQTLs (Fig. 3), belong to the fatty acid binding proteins (FABPs) family. This family of lipid chaperones exerts numerous functions highly related to lipid-mediated processes^35^.

**Figure 4:**
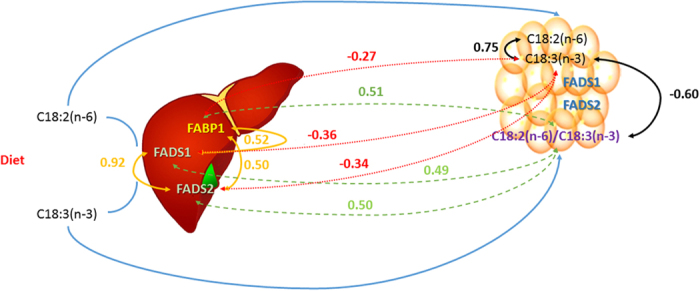
Schematic view of correlation values between liver mRNA expression of *FABP1, FADS1*, and *FADS2* and backfat composition of essential fatty acids (C18:2(n-6), C18:3(n-3) and C18:2(n-6)/C18:3(n-3)).

*FABP1* is highly expressed in the liver, but is also expressed in other tissues such as the intestine or kidney (reviewed in ref. 35). FABP1 has a high affinity for n-3 polyunsaturated fatty acids (PUFAs) and mediates the n-3-PUFA induction of PPARα target genes in fatty acid β-oxidation^36^. In fact, the *FABP1* gene ablation in mice inhibits long chain fatty acids (LCFA) oxidation, redirecting LCFA towards storage in adipose tissue and therefore promoting weight gain (reviewed in ref. 37). Moreover, *FABP1* has recently been suggested to be positively selected for meat quality in Berkshire pigs^38^. Notably in our study, *FABP1, FADS1* and *FADS2* mRNA expression were moderately inter-correlated between them (r_*FABP1-FADS1*_ = 0.52, p-value = 2.86 × 10^−09^; r_*FABP1-FADS2*_ = 0.50, p-value = 1.34 × 10^−08^) and also with the C18:2(n-6)/C18:3(n-3) fatty acid ratio in BF (r_*FABP1*_ = 0.51, p-value = 3.35 × 10^−08^; r_*FADS1*_ = 0.49, p-value = 1.36 × 10^−07^, r_*FADS2*_ = 0.50, p-value = 5.58 × 10^−08^) (Fig. 4). In mammals, *FADS1* and *FADS2* desaturases are required for the synthesis of highly unsaturated fatty acids (HUFAs) from essential fatty acids (C18:2(n-6) and C18:3(n-3)) supplied from the diet. HUFAs are essential for many physiological functions in animals (reviewed in ref. 39). Interestingly, a feedback regulation of HUFA synthesis is mediated by the transcriptional activation of desaturases by the antagonist transcription factors SREBP1c and PPARα^39^. The induction of desaturases by PPARα could help to ensure the availability of PUFAs which are necessary for their diverse functions^40^. Therefore, an increase in the mRNA levels of *FABP1* could indirectly activate the mRNA expression of desaturases, through the n-3 PUFAs mediated induction of PPARα and fatty acid oxidation. Supporting our hypothesis the mRNA expression of *SREBP1* and *PPARA* were moderately correlated with *FADS1* and *FADS2* mRNA expression (r_*FADS1-PPARA*_ = 0.48, p-value = 7.50 × 10^−08^; r_*FADS1-SREBP1*_ = 0.46, p-value = 3.35 × 10^−07^; r_*FADS2-PPARA*_ = 0.44, p-value = 1.16 × 10^−06^; r_*FADS2-SREBP1*_ = 0.53, p-value = 1.87 × 10^−09^). Furthermore, a functional interaction network between all of these genes was predicted by GeneMANIA^41^ (Supplementary Fig. S1).

The n-6/n-3 fatty acid profile is one of the healthfulness indicators of pork meat and many efforts have been made to increase n-3 fatty acids in pork by modifying the porcine diets^42^. Although C18:2(n-6) and C18:3(n-3) are essential fatty acids which are readily stored in adipose triglycerides (reviewed in ref. 39), our results support a genetic basis of phenotypic variation of these traits in pigs. Here, a major usage of n-3 PUFA in liver in animals with high mRNA levels of *FABP1* could explain the increase of BF n-6/n-3 PUFA ratio in these animals. In fact, slightly significant negative correlations between *FABP1* (r = −0.27, p-value = 5.49 × 10^−03^), *FADS1* (r = −0.36, p-value = 1.93 × 10^−04^) and *FADS2* (r = −0.34, p-value = 4.46 × 10^−04^) mRNA levels and BF C18:3(n-3) were observed (Fig. 4). Other factors such as the direct storage of C18:3(n-3) fatty acid in adipose triglycerides and desaturase activities in BF could be responsible of these low correlation values.

In the present study, the *cis*-SNP DIAS0001897 (p-value = 3.58 × 10^−6^, q-value = 0.007) was mapped in the exon 2 of *FABP1* determining a synonymous substitution, however it was not the most associated *cis*-SNP for the *FABP1* gene expression levels. Five markers (ALGA0019167, ALGA0019175, MARC0017363, MARC0054644 and CASI0010035) almost in complete linkage disequilibrium (LD) (D′ = 0.99) and surrounding the upstream and downstream *FABP1* genomic region (ENSSSCG00000008214, SSC3: 60,622,626–60,627,147 bp) were the most associated (p-value = 1.88 × 10^−8^, q-value = 0.00015; Supplementary Table S3) with the *FABP1* gene expression. Although this eQTL region is not overlapping with QTLs affecting meat quality traits in the BC1_LD population, further analysis is warranted due to the relevant role of this gene in the LCFA transport, specially ω3 fatty acids, and its subsequent usage in the organism.

On the other hand, the *FABP5* gene was located in a chromosomal region affecting IMFA composition in our animal population^10^. *FABP5* is highly expressed in epidermal cells of the skin, although it is also present in many other tissues including liver (reviewed in ref. 35). As additional members of the FABP family are also expressed in these tissues, the exact role of FABP5 is difficult to elucidate. Interestingly, in our study, *FABP1* and *FABP5* presented opposite patterns of expression with some genes such as the ELOVL members: *ELOVL6* and *ELOVL5* suggesting different roles in the functional pathways where those genes are implicated. Association analysis for *FABP5* mRNA expression in BF (unpublished data) and *Longissimus dorsi* muscle of BC1_LD animals were also performed^34^ but no association of *cis*-variants on *FABP5* gene expression in both tissues was evidenced, suggesting different mechanisms controlling *FABP5* gene expression among tissues. In our study, the most associated *cis*-variant affecting the *FABP5* mRNA levels in liver was the SNP MARC0115316 (p-value = 1.10 × 10^−7^, q-value = 0.001; Supplementary Table S3) located more than 700 kb upstream of *FABP5*.

Finally, four more genes presented associated *cis*-SNPs at chromosome level (q-value < 0.05): *ABCG8* on SSC3, *USF1* on SSC4, and *CYP2U1* and *HADH* on SSC8 (Supplementary Table S6). Remarkably, all these genes map in chromosomal regions associated with IMFA composition in our animal population^10^. Furthermore, *ABCG8* was also differentially expressed between individuals phenotypically extreme for IMFA composition^23^.

Overall, these results suggest that DNA variants in these regions may affect the expression levels of *ABCG8, CYP2U1, FABP5, HADH* and *USF1* and, for instance, may have a role in the determination of meat quality traits.

### *Trans*-eQTLs

A highly significant trans-eQTL associated with the *CROT* mRNA levels was identified on SSC13 (Table 1). The region spanned 12.4 Mb with a total of 33 eSNPs (Supplementary Table S3) which presented a high LD. CROT is a member of the carnitine acyltransferase enzymes which catalyze the exchange of acyl groups between carnitine and coenzyme A (CoA) having a crucial role in fatty acid oxidation^43^.

To go further in the characterization of the regulatory mechanisms that may be influencing *CROT* gene expression, gene annotation of this regulatory region was performed. Three genes related with lipid metabolism are located in this region: *Lipase H (LIPH*), a member of the mammalian triglyceride lipase family; *diacylglycerol kinase gamma-like (DGKG*), a member of the type I subfamily of diacylglycerol kinases that catalyzes the phosphorylation of the lipid second messenger diacylglycerol (DAG) to phosphatidic acid^44^; and *phosphatidylinositol-4, 5-bisphosphate 3-kinase catalytic subunit alpha (PIK3CA*), which encodes a catalytic subunit of the class IA phosphoinositide 3-kinases (PI3Ks). Interestingly, that gene is a key mediator of the hepatic insulin signaling, regulating glucose and lipid metabolism through the Akt/PKB pathway^45^. PI3Ks activates Akt/PKB which in turn phosphorylates *FoxO1* to inhibit the expression of the major gluconeogenic genes. In parallel, it also phosphorylates *PGC-1*α suppressing fatty acid oxidation in the liver^46^. Thus, this gene is a candidate gene to explain the variation in the mRNA levels of *CROT*.

Another significant eSNP (ASGA0093674, p-value = 1.94 × 10^−6^, q-value = 0.038; Supplementary Table S3) associated in *trans* with the expression of *PLA2G12A* was identified around 150 Mb on SSC2. Interestingly, the same region has been recently associated with the mRNA expression of the *PPARA* gene in the *Longissimus dorsi* muscle of BC1_LD^34^ suggesting a pleiotropic effect of this region in the expression of *PLA2G12A* and *PPARA* in liver and muscle, respectively.

PLA2G12A is an enzyme that belongs to the secreted phospholipase A2 (sPLA2) family. Phospholipases hydrolyse phospholipids generating a variety of lipid mediators^47^. Noteworthy, in this region different genes related to the lipid metabolism were mapped: *Rho GTPase activating protein 26 (ARHGAP26*), *fibroblast growth factor 1 (FGF1*), *histone deacetylase 3 (HDAC3*) and *nuclear receptor subfamily 3 group C member 1 (NR3C1*). Among them, *HDAC3* and *NR3C1* play a key role in transcriptional regulation. HDAC3 acts as a transcriptional repressor implicated in multiple hepatic signalling pathways and controlling the circadian rhythm of hepatic lipid metabolism^48^,^49^. NR3C1 can activate gene transcription (transactivation) directly binding in the promoters of genes related with glucose and fat metabolism, or can influence the activity of immune-regulating transcription factors without contacting DNA itself (transrepression)^50^. Notably, *PLA2G12A* has been identified as a potential NR3C1 primary target in 3T3-L1 adipocytes: its expression is modulated by glucocorticoids and it contains glucocorticoid receptor binding regions in its sequence^51^. Thus, *NR3C1* is a potential candidate gene to determine the mRNA variation of *PLA2G12A*.

Finally, at chromosome level, we identified the same chromosomal region on SSCX (X: 103,335,800–105,453,804 bp) associated in *trans* with the expression of *FADS1* and *FADS2* (Supplementary Fig. S2). In contrast, the expression of *FADS3* was associated with a chromosomal region on SSC6 (6: 39,526,829–39,546,925 bp) (Supplementary Fig. S2). These results agree with the high correlation observed between the mRNA expression levels of *FADS1* and *FADS2* (r = 0.92, p-value = 1.11 × 10^−17^) compared with the correlation between *FADS1, FADS2* and *FADS3* mRNA expression (r_*FADS1-FADS3*_ = 0.31, p-value = 7.90 × 10^−4^; r_*FADS2-FADS3*_ = 0.29, p-value = 1.62 × 10^−3^), which suggest a common element regulating *FADS1* and *FADS2* expression. In fact, the pig genomic structure of *FADS* gene family on SSC2 has been recently determined showing a similar organization to that in other mammals^52^. The *FADS1* and *FADS2* genes are oriented head-to-head and the proximity of their promoters suggests a co-ordinately controlled transcription by common regulatory mechanisms^39^. When the 103–105 Mb SSCX *trans*-regulatory region was analysed in detail a promising candidate gene that could affect the expression of desaturases was identified: the *acyl-CoA synthetase long-chain family member 4 (ACSL4*) gene. ACSL4 is an enzyme that belongs to the long-chain acyl-CoA synthetases (ACSLs) family which play a key role in the synthesis of cellular lipids and degradation of fatty acids via β-oxidation. Interestingly, ACSL4 preferentially utilizes arachidonic acid (AA; C20:4(n-6)) and eicosapentaenoic acid (EPA; C20:5(n-3)) as substrates^53^, two important products of the FADS1/FADS2 pathway that regulate *FADS1* and *FADS2* gene expression^54^,^55^. Thus, *ACSL4* is a strong candidate gene to regulate the expression of *FADS1* and *FADS2* through the use of AA and EPA as substrates. Remarkably, *ACSL4* has been described as a candidate gene for several QTLs, some of them affecting meat quality traits in the BC1_LD population^56^.

Overall, our results suggest that the *FABP1* and *ACSL4* genes may be playing an important role in the desaturase pathway regulation in liver and in the PUFAs composition determination both in liver and BF. Further studies are warranted to validate our hypothesis.

### Expression-based genome-wide association analysis revealed two hotspot regions on SSC8

In agreement with previous studies in which *trans*-eQTLs hotspots affecting the expression of many genes were identified^57^, two chromosomal intervals located on SSC8 and spanning 1.4 Mb (8:86,660,228–88,128,717) and 7.7 Mb (8:116,174,552–123,996,880) showed a significant signal associated with the expression of *CYP2U1* and *PPARA*, and *DGAT1, EGF* and *PPARA*, respectively (Table 1). A detailed analysis of this region at chromosomal level, considering the q-value ≤ 0.05, showed that, in addition to the four previous genes, associations with the hotspot at 86–88 Mb were observed for the expression of *DGAT1* and *HADH* (Table 1). Furthermore, the hotspot at 116–123 Mb was also associated at chromosome level with the expression of *AGPAT2, APOB, CYP2U1, ESRRA*, and *HADH* (Table 1). Notably, a GWAS performed in the BC1_LD population highlighted these two SSC8 regions as associated with the *Longissimus dorsi* IMFA composition^10^.

**Figure 5:**
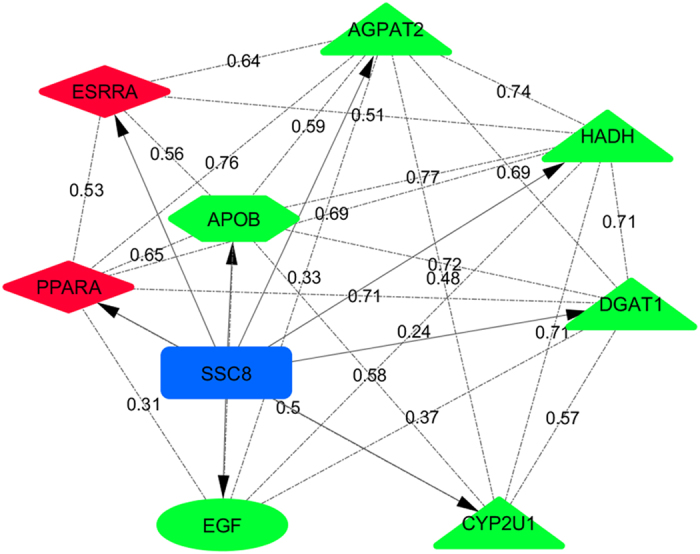
Co-expression network of genes associated with the *trans*-eQTL hotspots on SSC8 using PCIT algorithm. Red color indicates transcriptional factors and green color indicates the rest of genes identified in the SCC8 hotspots. Node shape represents epidermal growth factor (ellipse), lipid-related enzymes (triangle), transfer and transport enzymes (hexagon), transcriptional regulators (diamond) and *trans*-eQTL hotspots on SSC8 (rectangle).

In order to study the co-expression pattern of genes regulated for these eQTL intervals on SSC8, a liver weighted gene co-expression network was built using PCIT algorithm^26^. Significant positive correlations ranking from 0.24 to 0.77 were observed among genes regulated by the same eQTL (Fig. 5, Supplementary Table S7), suggesting a common regulatory direction/effect. The lowest co-expression values were observed for the *EGF* gene; this gene is mapped at 120,390,710–120,418,097 bp in the current *Sscrofa10*.*2* assembly suggesting the presence of an independent *cis*-factor regulating the expression of *EGF*.

Furthermore, gene annotation of these two hotspot regions was performed to find potential *trans*-acting genetic variants modulating the expression of the associated genes. Whereas no relevant genes were identified at the 86–88 Mb SSC8 hotspot region, three SNPs in the *TBCK* gene (Ensembl Id: ENSSSCG00000021784) codifying for a TBC1 domain containing kinase and located in the 116–123 Mb SSC8 hotspot region were associated in *trans* with the expression of all of the genes, with the exception of *CYP2U1, EGF*, and *HADH*.

Protein kinases play a key role in many cellular processes including metabolism and transcription among others^58^. TBCK has been classified as a pseudokinase because it lacks some subdomains of the kinase domain required for kinase activity^58^,^59^. Although pseudokinases were predicted to be inactive, some studies demonstrated the important role of these proteins in regulating fundamental cellular processes (review in refs 60 and 61). In fact, Liu *et al*.^59^ recently determined that knockdown of TBCK significantly decreased the protein and mRNA levels of mTORC components altering the mTOR signaling pathway. Interestingly, mTOR signaling regulates lipid homeostasis through the control of key regulators of hepatic lipid metabolism such as PPARA and/or SREBF1. Inhibition of hepatic mTORC1 and mTORC2 impaired lipid metabolism decreasing lipid synthesis, lipid uptake and increasing lipolysis^32^. Remarkably, the SSC8 hotspot was associated with the *PPARA* expression phenotype, a key transcription factor that regulates the expression of lipogenic genes (Fig. 5). Accordingly, iRegulon^62^ identified the PPARA (NES = 8.183) as an enriched TF that can bind all the genes located within the SSC8 hotspot with the exception of *APOB*.

Finally, to assess the existence of literature reported direct functional interactions between *TBCK* and genes associated with the SSC8 hotspot, we constructed gene functional network using GeneMANIA. The resulted network showed interactions between *TBCK* and *APOB, CYP2U1*, and *PPARA*. After measuring with RT-qPCR the liver mRNA expression of *TBCK* in our samples, these predicted associations were validated through moderate correlations between *TBCK* and *APOB* (r = 0.42, p-value = 1.01 × 10^−5^), *CYP2U1* (r = 0.51, p-value = 3.73 × 10^−8^) and *PPARA* (r = 0.43, p-value = 8.38 × 10^−6^).

Taken together, these results suggest a possible direct role of *TBCK* in lipid metabolism and point out the *TBCK* as a promising candidate gene affecting the expression of genes associated with the hotspot on SSC8. Unfortunately, the *TBCK* gene is not well mapped in the current *Sscrofa10*.*2* assembly, at least there are three Ensembl ID codifying for different exons of *TBCK* (ENSSSCG00000022709, ENSSSCG00000009154, ENSSSCG00000021784). In human, this gene has 27 exons with multiple isoforms (ENSG00000145348). For that reason, further genetic and functional analyses are necessary to determine the possible regulatory role of *TBCK* in these lipid expression phenotypes, and, for instance, in the genetic determination of fatty acid composition traits. Finally, we cannot discard that other genetic variants in LD with *TBCK* might control the expression of all the genes associated with the hotspot on SSC8 including *TBCK*. In this SSC8 region, other interesting genes related with lipid metabolism were also mapped: *ELOVL6, PLA2G12A* and *SGMS2*. Although eGWAs analyses were also performed for *ELOVL6* and *PLA2G12A* expression phenotypes, we did not detect any significant associated signal in the SSC8 hotspot.

### Positional concordance between eSNPs and fat related QTLs

Finally, to verify whether or not the identified 92 eSNPs overlapped with QTL associated with meat quality-related traits, we mapped them against QTLs up to 30 Mb in size of the Pig QTLdb (Release 29, Apr 29, 2016). It should be noted that 36.9% (34/92) of the eSNPs overlapped with a total of 68 QTLs of which 29 QTLs were fatness traits and 14 QTLs were fat composition traits (Supplementary Table S8). It is noteworthy that, as we described before, more of the eSNPs reported in the present study fell into QTL intervals for fatty acid composition traits in an Iberian x Landrace cross^10^,^11^,^12^,^63^,^64^ which agrees with the fact that most of the trait-associated SNPs are significantly more likely to be eQTLs^65^. Thus, these SNPs may play an important role in the regulation of key genes for the lipid metabolism in liver, one of the principal organ controlling whole-body energetic homeostasis and for instance with an important effect in the lipid composition of other organs such as backfat and muscle.

## Conclusions

In this study, gene co-expression and eGWAs analyses were applied to detect DNA variants associated with expression phenotypes of 43 lipid related-genes. Co-expression analysis revealed sex-dimorphism associated with hepatic lipid metabolism. Also, we reported a list of candidate genes and genetic networks that may affect the expression of lipid-related genes and may determine changes in fat content and fatty acid composition traits. Although further genetic and functional studies are necessary to validate our findings, these results enhance our knowledge of the regulatory mechanisms implicated in liver lipid metabolism and provide new insights into the understanding of molecular mechanisms implicated in lipid related traits.

## Methods

### Animal Material

The population studied was generated by crossing three Iberian (Guadyerbas line) boars with 31 Landrace sows^63^. Subsequently, five F1 males were backcrossed with 26 Landrace sows (BC1_LD). Here, we report results based on 111 BC1_LD (25% Iberian x 75% Landrace) pigs (64 females and 47 males) from 26 full-sib families. Animals were fed *ad libitum* with a cereal-based commercial diet and slaughtered at an average age of 179.8 ± 2.6 days. Animal care and procedures were performed according Spanish and European regulations about the protection of animals used in experimentation, following national and institutional guidelines for the Good Experimental Practices and approved by the Ethical Committee of the Institution (IRTA- Institut de Recerca i Tecnologia Agroalimentàries). Samples of liver were collected, snap frozen in liquid nitrogen and stored at −80 °C. Genomic DNA was extracted from blood samples according to the phenol-chloroform method^66^.

### Expression analysis

Total RNA was obtained from liver using the RiboPure kit (Ambion), following the manufacturer’s recommendations. RNA was quantified using the NanoDrop ND-1000 spectrophotometer (NanoDrop products) and the RNA integrity was assessed by Agilent Bioanalyzer-2100 (Agilent Technologies). One microgram of total RNA was reverse-transcribed into cDNA using the High-Capacity cDNA Reverse Transcription kit (Applied Biosystems) in 20 μl of reactions, following the manufacturer’s instructions.

A total of 48 genes (44 target genes and 4 reference genes) were analysed by RT-qPCR. Target genes were lipid-related genes selected based on previous studies of our group in which GWAS and liver transcriptome analyses highlighted candidate genes associated with IMFA composition in the BC1_LD population^10^,^23^. In addition, the list was completed adding genes with different roles in liver lipid metabolism.

All the primers used in this study were designed using PrimerExpress 2.0 software (Applied Biosystems) and are shown in Supplementary Table S9. Prior to perform the Fluidigm Real-Time PCR, all the assays were tested for PCR specificity in an ABI PRISM 7900 Sequence Detection System (Applied Biosystems) using two-fold dilutions (1/20, 1/200) of a pool of ten cDNA samples and a minus RT control to check the presence of DNA contamination. Melting curve analysis was performed for all the assays.

The 48.48 microfluidic dynamic array IFC chip (Fluidigm) was used to analyse the expression of 48 genes (44 target genes and 4 reference genes) of 111 backcross animals following a protocol previously described^67^. Data was collected using the Fluidigm Real-Time PCR analysis software 3.0.2 (Fluidigm) and analysed using the DAG expression software 1.0.4.11^68^ applying the relative standard curve method. Standard curves with a four-fold dilutions series (1/4, 1/16, 1/64, 1/256, 1/1024) per triplicate of a pool of 10 cDNA samples were constructed for each gene to extrapolate the quantity values of the studied samples. Samples targeted in our study were analysed in duplicate. A dissociation curve was drawn for each primer pair. The PCR efficiencies were almost 90% for all the assays with low coefficient of inter-assay variation (<1.9%). Of the four endogenous genes tested (*ACTB, B2M, HPRT1* and, *TBP*), *ACTB* and *TBP* were the genes with the most stable expression and were selected as reference controls. The normalized quantity (NQ) values of each sample and assay were used to compare our data. Data obtained were normalized by performing log_2_ transformation of the NQ value. The sex effect was also tested by using a linear model in R^69^.

### Genotyping, quality control and expression-associated analysis

A total of 197 animals were genotyped for 62,163 SNPs with the Porcine SNP60K BeadChip^70^ and using the Infinium HD Assay Ultra protocol (Illumina Inc.; San Diego, USA)^11^. Plink software^71^ was used to remove markers that showed a minor allele frequency (MAF) less than 5% and SNPs with more than 5% of missing genotypes. The SNPs not mapped in the *Sscrofa10*.*2* assembly were also excluded. After quality control a subset of 40,502 SNPs were retained for subsequent analysis.

In order to detect expression-associated SNPs (eSNPs), GWAS was performed using as phenotype the expression pattern of 43 genes in liver tissue. A mixed model accounting for additive effects (see below) was performed using Qxpak 5.0^72^:


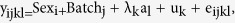


in which y_ijkl_ is the k^th^ individual gene expression, sex (two levels) and batch (five levels) are fixed effects, λ_k_ is a −1, 0, +1 indicator variable depending on the k^th^ individual genotype for the l^th^ SNP, a_l_ represents the additive effect associated with SNP, u_k_ represents the infinitesimal genetic effect treated as random and distributed as N (0, **A**σ^2^_u_) where **A** is a numerator of kinship matrix and e_ijkl_ is the residual.

To correct for multiple testing, the false discovery rate (FDR) was calculated with the R package q-value^73^ and the cut-off of the significant association was set at q-value ≤ 0.05. A more stringent q-value of ≤0.01 was applied for *CROT, FABP1* and *FABP5* to focus our analysis on the most associated SNPs at the signal peak.

### Gene annotation, functional classification and gene network analysis

The significantly associated eSNPs were mapped in the *Sscrofa10*.*2* assembly. Gene annotations were retrieved from the Ensembl Genes 80 Database using the Biomart software^74^, considering 1 Mb downstream/upstream of around the candidate chromosomal regions.

The SNPs identified were classified as *cis* when they were located within 1 Mb from the gene analysed and as *trans* when they were located elsewhere in the genome. Due to the high LD in our population, the number of significant SNPs belonging to the same interval was considered among associated SNPs less than 10 Mb apart.

The *Core Analysis* function included in the Ingenuity Pathway Analysis software (IPA; Ingenuity Systems) was applied to interpret data in the context of biological processes, pathways and networks. All information generated in this software is based on the Ingenuity Pathway Knowledge Base (IPKB), which is derived from known functions and interactions of genes published in the literature. Furthermore, GeneMANIA^41^ was used to explore the interaction and networks between genes.

### Co-expression and functional analysis

Liver weighted gene co-expression networks were built using PCIT algorithm. This algorithm implements first-order partial correlation coefficients combined with an information theory approach to identify meaningful gene–gene interactions^26^,^75^. Before analyzing, gene expression was normalized and then adjusted for fixed effects (sex with the exception of sex-specific networks and batch) with the linear model procedure of R software^69^. Cytoscape software^76^ was used to visualize the inferred gene networks. In all cases, the cut-off for considering a significance overrepresentation was established by multiple testing correction of the p-value (FDR < 0.05). Finally, specific node centrality values and network topological parameters were calculated using CentiScaPe plugin^77^.

The iRegulonv1.3 Cytoscape plugin^62^ was used to *in silico* identify common transcription-factor binding sites motifs.

### Correlation of gene expression and phenotypes

Pairwise correlations among gene expression and FA composition percentages in muscle^10^ and backfat^11^ were carried out to explore the relationships between gene expression and phenotypes. All values were normalized applying the log_2_ of raw data if necessary. Afterwards, gene expression was corrected by sex (two levels) and batch (five levels) effects, whereas FA composition percentages were corrected by sex, batch and carcass weight. The remaining residuals of the phenotypes and gene-expression values corrected for the corresponding effects were used to obtain the pairwise correlations.

## Additional Information

**How to cite this article:** Ballester, M. *et al*. Integration of liver gene co-expression networks and eGWAs analyses highlighted candidate regulators implicated in lipid metabolism in pigs. *Sci. Rep.* **7**, 46539; doi: 10.1038/srep46539 (2017).

**Publisher's note:** Springer Nature remains neutral with regard to jurisdictional claims in published maps and institutional affiliations.

## Supplementary Material

Supplementary Material

Supplementary Dataset 1

Supplementary Dataset 2

## Figures and Tables

**Table 1:** Description of the seven chromosomal regions associated with gene expression phenotypes.

Chr	SNP_Start	SNP_End	N SNPs	Position Mb Start-End	Associated_Trait	Size (Mb)	Hotspot SSC8 at chromosome level	Cis/Trans-eSNPs
2	ASGA0093674		1	150	PLA2G12A			*trans*
3	INRA0010653	MARC0027326	23	58–62.5	FABP1	4.5		*cis*
4	ALGA0025112	ALGA0025344	10	60–65.9	FABP5	4.2		*cis*
4	ASGA0019920	DIAS0001337	8	69.7–74.2	FABP5	4.5		*trans*
8	MARC0041739	ASGA0039160	7	86.7–88.1	CYP2U1/PPARA	1.40	DGAT1/HADH	*trans*
8	CASI0006714	DRGA0008764	10	116.2–123.9	DGAT1/EGF/PPARA	7.70	AGPAT2/APOB/CYP2U1/ESRRA/HADH	*trans*
13	ALGA0071744	DIAS0003262	33	122.3–134.7	CROT	12.4		*trans*
