# Supplementary material for: Integration of liver gene co-expression networks and eGWAs analyses highlighted candidate regulators implicated in lipid metabolism in pigs
Source: Sci Rep. 2017 Apr 19;7:46539. doi: 10.1038/srep46539 (PMC5396199; doi:10.1038/srep46539)
Supplement: Supplementary Material [file srep46539-s1.pdf]

SUPPLEMENTARY MATERIAL

Integration of liver gene co-expression networks and eGWAs analyses highlighted candidate regulators implicated in lipid metabolism in pigs

Maria Ballester[1,2,3*], Yuliaxis Ramayo-Caldas[1,2,4], Manuel Revilla[1,2], Jordi Corominas[1,2], Anna Castelló[1,2], Jordi Estellé[4], Ana I. Fernández[5], Josep M. Folch[1,2]

[1]Departament de Ciència Animal i dels Aliments, Facultat de Veterinària, Campus UAB, Bellaterra, 08193, Barcelona, Spain

[2]Plant and Animal Genomics, Centre de Recerca en Agrigenòmica (Consorci CSIC-IRTA-UAB-UB), Edifici CRAG, Campus UAB, Bellaterra, 08193, Barcelona, Spain

[3]IRTA, Genètica i Millora Animal, Torre Marimon, 08140 Caldes de Montbui, Spain

[4]Génétique Animale et Biologie Intégrative, Institut National de la Recherche Agronomique, AgroParisTech, Université Paris-Saclay, Jouy-en-Josas, France

[5]Departamento de Mejora Genética Animal, INIA, Ctra. de la Coruña km. 7, 28040, Madrid, Spain

[*]E-mail: maria.ballester@irta.cat

Figure S1. Evidence of interactions between *FABP1, FADS1, FADS2, PPAR*α and *SREBP1c* using GeneMANIA.

Figure S2. Plot of GWAS for *FADS1, FADS2* and *FADS3* gene expression in liver. Association analysis between the liver *FADS1* (A), *FADS2* (B) and *FADS3* (C) expression level and genotypes of markers included in the Porcine SNP60 Bead-Chip (Illumina). The X-axis represents the chromosomes, and the Y-axis shows the –log10 (p-value).

Table S1. Mean comparison of liver gene expression levels of 43 lipid-related genes between males and females.

Table S2. Topological mean parameters of male and female liver co-expression networks.

Table S3. Description of the 92 significant expression-associated SNPs (eSNPs).

Table S4. List of genes annotated in the seven chromosomal regions associated with gene expression phenotypes.

Table S5. Top-five associated networks and molecules from the list of genes annotated in the seven eQTLs based on IPA

Table S6. Description of the 17 significant *cis*-eSNPs at chromosome level.

Table S7. Significant co-expression values among genes regulated by the hotspots on SSC8.

Table S8. Positional concordance among the 92 eSNPs and QTLs deposited in the pig QTL database for fatness and fat composition related traits.

Table S9. List of primers for the RT-qPCR analysis.

Figure S1. Evidence of interactions between *FABP1*, *FADS1*, *FADS2*, *PPAR*α and *SREBP1c* using GeneMANIA.

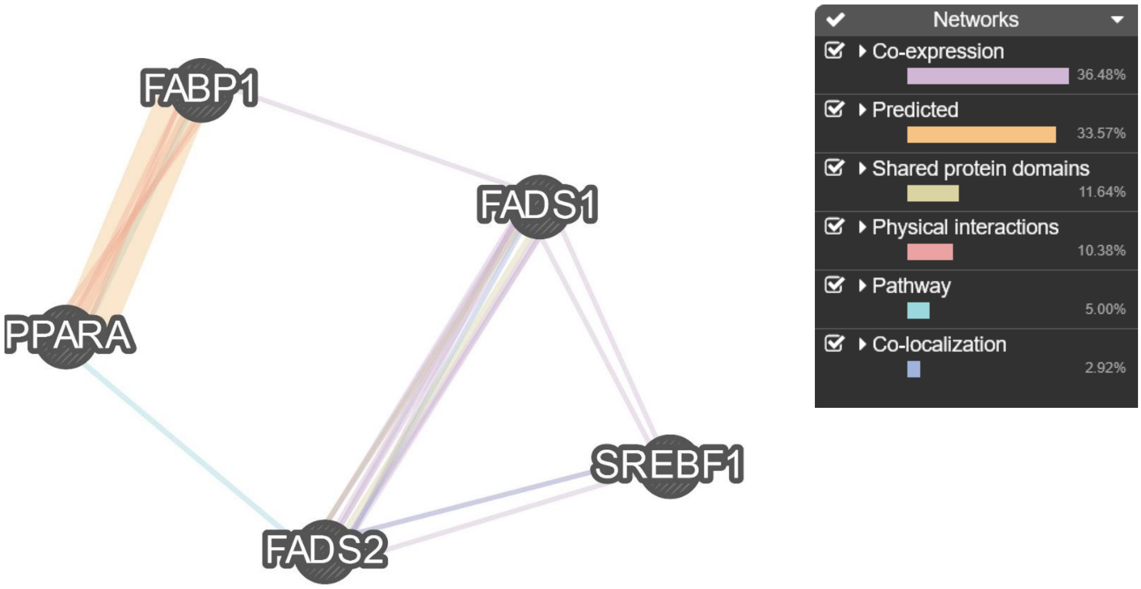

Figure S2. Plot of GWAS for *FADS1*, *FADS2* and *FADS3* gene expression in liver. Association analysis between the liver *FADS1* (A), *FADS2* (B) and *FADS3* (C) expression level and genotypes of markers included in the Porcine SNP60 Bead-Chip (Illumina). The X-axis represents the chromosomes, and the Y-axis shows the –log10 (p-value).

(A)

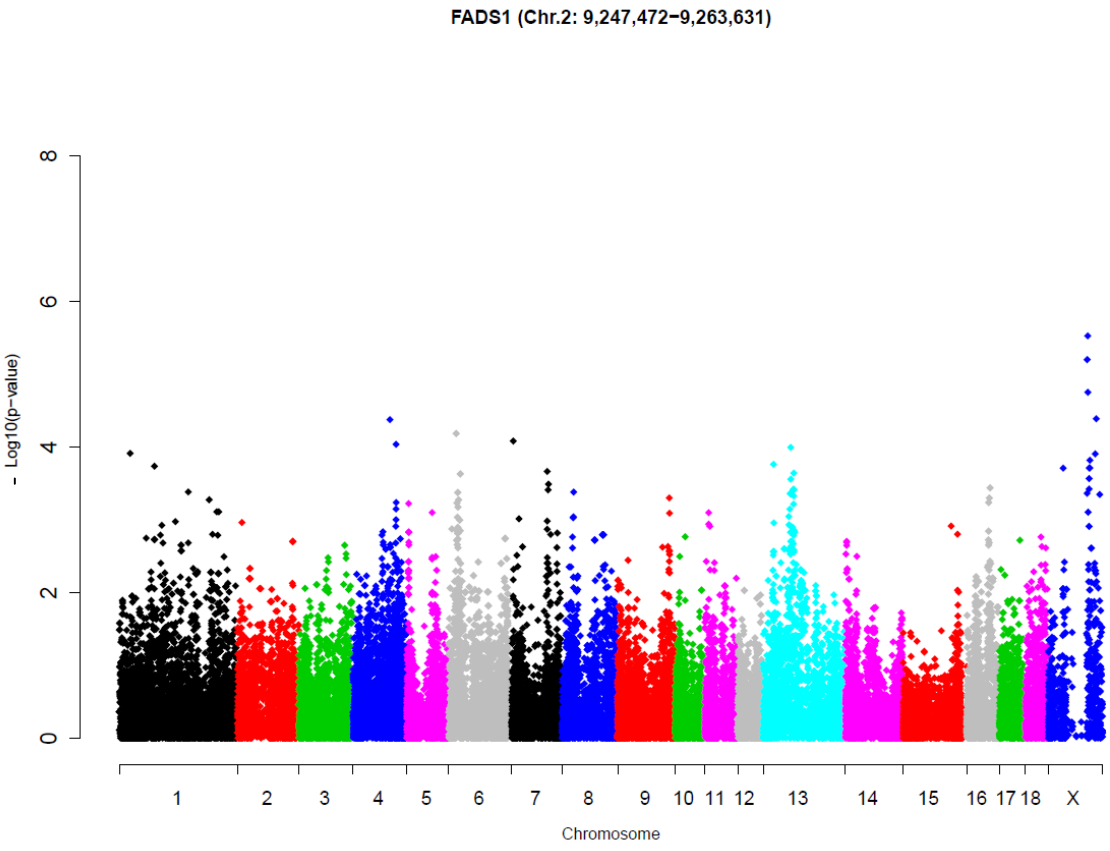

(B)

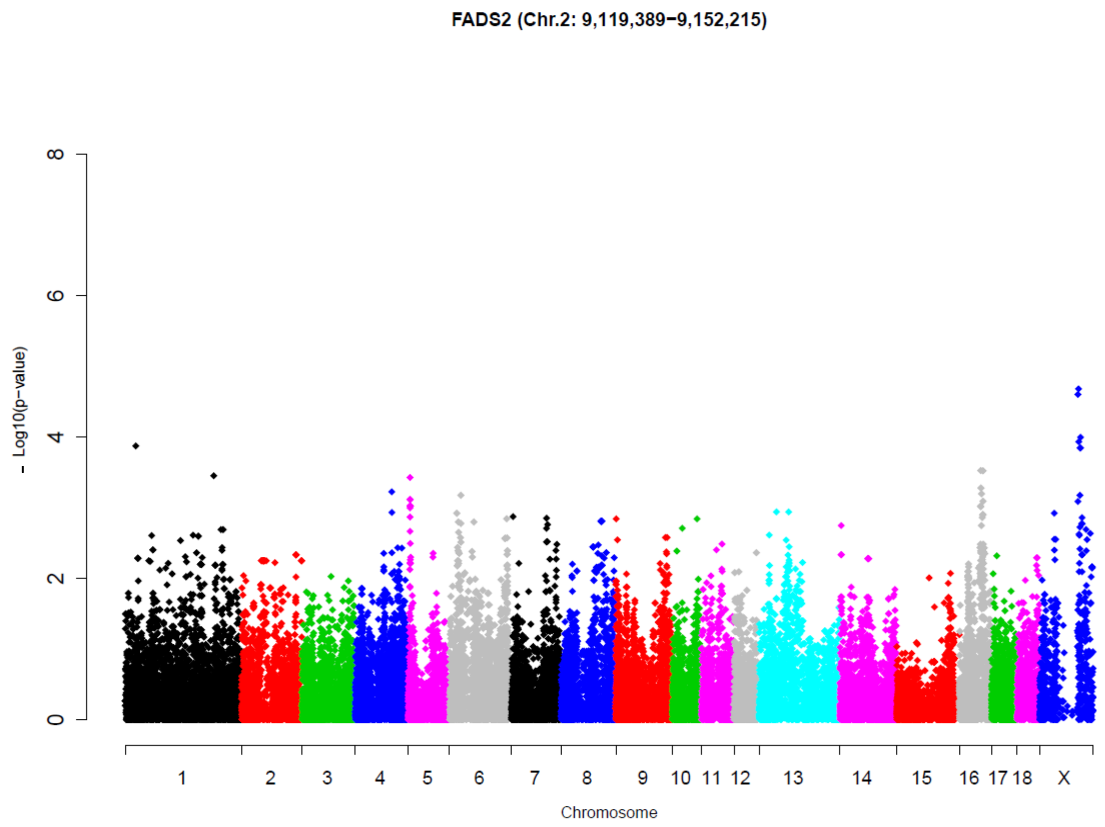
FADS2 (Chr.2: 9,119,389−9,152,215)
- Log10(p−value)
0
2
4
6
8
1 2 3 4 5 6 7 8 9 10 11 12 13 14 15 16 17 18 X
Chromosome

(C)

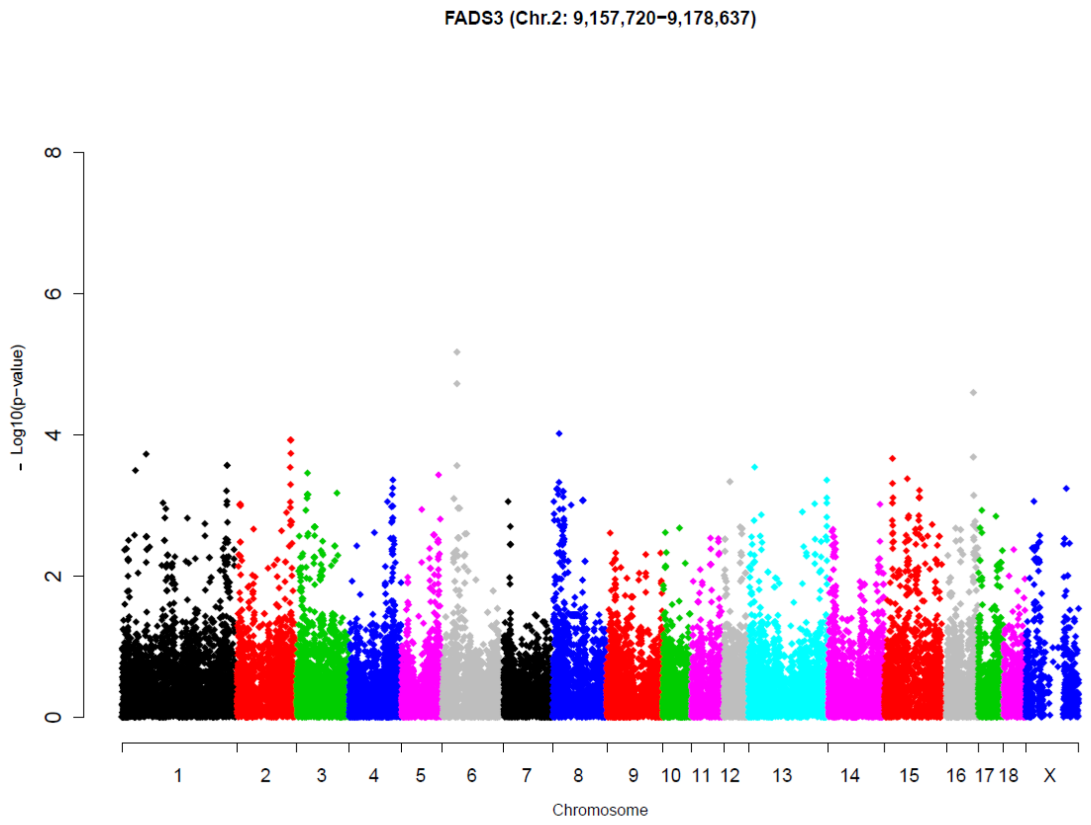
FADS3 (Chr.2: 9,157,720−9,178,637)
- Log10(p−value)
0
2
4
6
8
1 2 3 4 5 6 7 8 9 10 11 12 13 14 15 16 17 18 X
Chromosome

Table S1. Mean comparison of liver gene expression levels of 43 lipid-related genes between males and females.

Gene	Mean in Male	Mean in Female	P-value
ABCG8	1.10638507	1.32735359	4.05E-004
ACSM5	0.79516155	1.1851219	2.22E-010
AGPAT2	1.19071574	1.38563175	5.07E-003
APOA2	0.20550265	0.02713742	2.41E-001
APOB	-0.02306437	0.11954847	1.32E-002
ARNT	0.79698145	1.05437107	3.40E-006
CPT1A	0.29427247	0.16898876	3.37E-001
CROT	0.26698294	-0.66364779	1.47E-008
CYP2U1	0.82864276	1.03923552	8.59E-003
CYP7A1	-2.44494517	-0.05319579	4.03E-009
DGAT1	-0.41921145	0.36465435	1.30E-015
EGF	-1.22717633	-0.85612796	3.78E-004
ELOVL5	1.06074307	1.23407169	1.00E-002
ELOVL6	0.73797252	0.84591396	5.52E-002
ESRRA	0.34666028	0.30251565	3.73E-001
FABP1	-0.59242455	-0.57381859	9.66E-001
FABP5	-0.92984332	-0.72534364	2.05E-001
FADS1	-1.72876913	-0.72295389	2.02E-006
FADS2	-1.47984864	-0.29575868	1.75E-008
FADS3	-0.10099681	0.17610714	1.28E-004
FOXA1	0.22632908	0.4291075	2.08E-002
HADH	0.06495191	0.22201133	4.85E-002
HNF4A	1.09180931	1.17050052	1.65E-001
HNF4G	0.7645411	0.83374403	1.63E-001
KLF10	0.03005835	0.13973121	2.20E-001
LIPC	0.86223939	1.08434376	1.88E-006
LPIN1	-0.56350028	0.17233961	2.53E-006

MLXIPL	1.00207993	1.51263934	5.10E-007
MTTP	0.71398821	0.74801256	1.22E-001
NFKB1	0.87144002	0.97034133	2.84E-005
NR1H3	0.32850425	0.49924015	3.74E-003
PLA2G12A	0.94732107	0.97141924	3.61E-001
PLCB2	-0.02181955	0.32819592	9.73E-006
POU2F1	0.08581884	0.22957523	3.26E-002
PPAP2A	-0.19031833	0.0091592	1.51E-004
PPARA	0.87208797	1.08429168	7.95E-005
PPARD	0.41621572	0.26473171	1.91E-001
PPARGC1A	-0.14427855	0.01644963	1.71E-002
PEX2	-0.07879949	0.30253662	1.34E-005
SCAP	0.34398852	0.64865395	2.06E-005
SCD1	-0.34087252	0.32686898	5.34E-005
SREBF1	-0.72304021	0.36053337	3.08E-012
USF1	1.11536894	1.16965193	9.58E-002

Table S2. Topological mean parameters of male and female liver co-expression networks.

Topological parameters	Female	Male
Average Distance	1.76655052	1.45876887
Centroid mean value	-12.5952381	-11.5
Closeness mean value unDir	0.01406093	0.01705652
Degree mean value	14.2380952	22.9047619
Eccentricity mean value	0.34126984	0.4484127
Radiality mean value	3.23344948	2.54123113
Stress mean value	251.380952	201.666667

Table S4. List of genes annotated in the seven chromosomal regions associated with gene expression phenotypes.

**Gene list**				
ABCC5	DGKG	LARP7	POU4F2	VPS8
ABCE1	DIAPH1	LEF1	PPA2	VWA5B2
ABCF3	DKK2	LIPH	PPP1R42	XKR9
ACTL6A	DNAJB11	LRIT3	PRDM14	YEATS2
ADHFE1	DNAJC19	LSM6	PREX2	ZMAT3
AHSG	DNAJC5B	MAGEF1	PRMT9	ZNF827
AIMP1	DVL3	MAP3K13	PSMD2	
ALG3	ECE2	MASP1	RELL2	
ALPK1	EDNRA	MCCC1	RFC4	
ANAPC10	EIF2B5	MCF2L2	RNF14	
ANK2	EIF4A2	MCMDC2	RPL34	
AP1AR	EIF4G1	MMAA	RRH	
AP2M1	ELOVL6	MRPL47	RRS1	
ARAP3	ENPEP	MSC	RTP1	
ARFGEF1	EPHB3	MTFR1	RTP2	
ARHGAP10	ETNPPL	MYBL1	SEC24B	
ARHGAP26	EYA1	NAALADL2	SENP2	
ARHGEF38	FAM131A	NCOA2	SGK3	
ARMC1	FCHSD1	NDFIP1	SGMS2	
ARSJ	FETUB	NDUFB5	SLC10A7	
ATP11B	FGF1	NLGN1	SLCO5A1	
B3GNT5	FXR1	NPNT	SOX2	
BCL6	GAR1	NR3C1	SPRY4	
C8orf34	GNPDA1	NR3C2	SST	
C8orf46	GSTCD	OSTC	SULF1	
CALM1	HADH	OTUD4	TAF7	
CAMK2D	HDAC3	PARL	TBCCD1	
CAMK2N2	HHIP	PCDH1	TBCK	
CASP6	HRG	PCDH12	TBL1XR1	
CCDC109B	HTR3C	PCDHB16	THPO	
CCDC39	HTR3D	PCDHGA12	TIFA	
CFI	IGF2BP2	PCDHGA8	TMEM184C	
CHRD	INTS12	PCDHGA9	TMEM41A	
CLCN2	KCNMB2	PCDHGB5	TRAM1	
COPS5	KCNMB3	PCDHGC5	TRIM55	
CPA6	KIAA0141	PDE7A	TRPA1	
CRH	KLHL24	PEX5L	TTC14	
CRYGS	KLHL6	PIK3CA	TTC29	
CSPP1	KNG1	PITX2	UGT8	
CYP2U1	LACTB2	PLA2G12A	USP13	
DCUN1D1	LAMP3	POLR2H	VCPIP1	

Table S5. Top-five associated networks and molecules from the list of genes annotated in the seven eQTLs based on IPA.

ID	Molecules in Network	Score	Focus Molecules	Top Diseases and Functions
1	Akt,ARAP3,ARFGEF1,C8orf44-SGK3/SGK3,CASP6,caspase,CPA6,DIAPH1,DNAJB11,DNAJC19,DNAJC5B,EIF2B5,EPHB3,EYA1,FABP1,GTPase,Hdac,HSP,Hsp70,Hsp90,Hsp22/Hsp40/Hsp90,KIAA0141,LSM6,Mlc,MSC,NDFIP1,PITX2,PP2A,PRDM14,PREX2,SEC24B,SOX2,TRIM55,trypsin,USP13	45	24	Cellular Development, Digestive System Development and Function, Auditory and Vestibular System Development and Function
2	AHSG,ANK2,C/ebp,CAMK2N2,Cbp/p300,Creb,CRH,Cyclin A,DKK2,ELOVL6,ERK1/2,estrogen receptor,FGF1,Fgfr,HDAC3,HDL,histone deacetylase,Histone h4,LEF1,LIPH,N-cor,NCOA2,NCoR/SMRT corepressor,NLGN1,Nr1h,NR3C2,PCDH1,Pias,RNF14,SPRY4,SULF1,TBL1XR1,TTC29,UGT8,Wnt	35	20	Organismal Development, Endocrine System Disorders, Gastrointestinal Disease
3	AIMP1,Alpha Actinin,Ap1,ATP11B,B3GNT5,CAMK2D,Caspase 3/7,CD3,CHRD,DVL3,ENPEP,FABP5,FETUB,Fibrinogen,HRG,IgG,IKK (complex),IL1,KNG1,MAP3K13,Mapk,Mek,MYBL1,NFkB (complex),p70 S6k,p85 (pik3r),PDGF BB,PIK3CA,Ras,SENP2,Sos,Tgf beta,THPO,TIFA,TRPA1	31	18	Post-Translational Modification, Cellular Growth and Proliferation, Hematological System Development and Function
4	26s Proteasome,Actin,ACTL6A,ADCY,ARMC1,CALM1 (includes others),Calmodulin,Cg,CLCN2,EIF4A2,EIF4G1,ERK,FXR1,GNRH,HADH,HISTONE,Histone h3,Insulin,MEF2,NR3C1,PI3K (complex),Pka,Pka catalytic subunit,PLC,POLR2H,Proinsulin,PSMD2,RFC4,RNA polymerase II,RRS1,SST,STAT5a/b,TAF7,voltage-gated calcium channel,YEATS2	26	16	Drug Metabolism, Endocrine System Development and Function, Lipid Metabolism
5	ANKRD39,APP,Aspartyl Protease,CALML4,CCDC39,CFDP1,CUL3,DNAJC4,ETNPPL,HTT,INTS12,KCNC4,KCNMA1,KCNMB2,KCNMB3,KCNMB4,KCNU1,KCTD18,LARP7,MASP1,MTFR1,NAALADL2,ODF2L,PEX5L,PIN1,PPA2,PRPF40B,PRRC1,Rbfox3,RPL34,RPL3L,TBCK,WDR91,ZMAT4,ZNF706	20	13	Cell-To-Cell Signaling and Interaction, Nervous System Development and Function, Cardiac Arrythmia

Table S6. Description of the 17 significant *cis*-eSNPs at chromosome level.

Chr	SNP	bp	fA1	fA2	p-value	q-value	Associated_Gene	Consequence	Ensembl_GeneId	GeneId
3	ALGA0020228	102155060	0.79	0.21	2.82E-05	1.52E-02	ABCG8	intergenic_variant		
3	ASGA0015479	102763903	0.79	0.21	2.82E-05	1.52E-02	ABCG8	intron_variant	ENSSSCG00000008453	ABCG8
3	M1GA0004530	103046883	0.79	0.21	2.82E-05	1.52E-02	ABCG8	intron_variant	ENSSSCG00000028599	orthologhe THADA
3	MARC0037203	102111329	0.87	0.13	6.62E-05	2.04E-02	ABCG8	intergenic_variant		
3	H3GA0010157	102401629	0.87	0.13	6.62E-05	2.04E-02	ABCG8	intron_variant	ENSSSCT00000009253	PPM1B
3	H3GA0010212	104427661	0.72	0.28	1.49E-04	4.02E-02	ABCG8	intergenic_variant		
3	ALGA0020335	104692463	0.71	0.29	1.96E-04	4.70E-02	ABCG8	intergenic_variant		
4	ASGA0020802	97973034	0.74	0.26	8.20E-06	2.07E-02	USF1	upstream_gene_variant	ENSSSCG00000006383	VANGL2
8	ALGA0049276	122249328	0.93	0.07	3.83E-06	3.72E-03	CYP2U1	intron_variant	ENSSSCG00000009150	HADH
8	ALGA0110177	122654894	0.41	0.59	6.02E-05	1.58E-02	HADH	intergenic_variant		
8	ASGA0106126	122655115	0.41	0.59	6.02E-05	1.58E-02	HADH	intergenic_variant		
8	ASGA0103857	122655547	0.41	0.59	6.02E-05	1.58E-02	HADH	intergenic_variant		
8	MARC0003202	122664524	0.41	0.59	6.02E-05	1.58E-02	HADH	intergenic_variant		
8	ALGA0108851	122072309	0.42	0.58	9.28E-05	1.95E-02	HADH	intergenic_variant		
8	ASGA0089440	124212852	0.46	0.54	1.73E-04	2.98E-02	HADH	intron_variant	ENSSSCG00000020821	NPNT
8	ALGA0049269	121743168	0.42	0.58	2.48E-04	3.62E-02	HADH	intergenic_variant		
8	MARC0037703	122288920	0.61	0.39	2.88E-04	3.78E-02	HADH	intron_variant	ENSSSCG00000009151	CYP2U1

Table S7. Significant co-expression values among genes regulated by the hotspots on SSC8.

From	To	Weight	sign
APOB	HADH	0.77372545	+
AGPAT2	PPARA	0.75664717	+
AGPAT2	HADH	0.73869884	+
DGAT1	APOB	0.724836052	+
CYP2U1	HADH	0.716647029	+
DGAT1	PPARA	0.71526891	+
DGAT1	HADH	0.711374998	+
PPARA	HADH	0.69495374	+
AGPAT2	DGAT1	0.688199759	+
PPARA	APOB	0.654403329	+
AGPAT2	ESRRA	0.635816097	+
AGPAT2	APOB	0.59262836	+
CYP2U1	APOB	0.580422282	+
CYP2U1	DGAT1	0.57343328	+
ESRRA	APOB	0.559513271	+
ESRRA	PPARA	0.530570805	+
ESRRA	HADH	0.50790447	+
CYP2U1	PPARA	0.499788463	+
CYP2U1	AGPAT2	0.484627783	+
DGAT1	EGF	0.365692794	+
AGPAT2	EGF	0.325705379	+
EGF	PPARA	0.309143394	+
EGF	APOB	0.240296617	+
EGF	HADH	0.240153566	+

Table S9. List of primers for the RT-qPCR analysis.

Gene	Full name	Type	Primer sequence	
			Forward	Reverse
ABCG8	ATP binding cassette subfamily G member 8	Target	ATGTCCGCCCTCTTGTTCAT	TCCGAGTGGCATTTGGAGAT
ACSM5	acyl-CoA synthetase medium-chain family member 5	Target	TGTAATCTGTGCCAATCCCAAA	CATCATCTACGATCTGCACCTCAT
ACTB	actin beta	Endogenous	CAAGGACCTCTACGCCAACAC	TGGAGGCGCGATGATCTT
AGPAT2	1-acylglycerol-3-phosphate O-acyltransferase 2	Target	CATGGTCAGGGAGAAGCTCAA	GCCAGGTAGAAGGCACCTTTC
APOA2	apolipoprotein A2	Target	AACTTCAAACTCAGGCCAAGACTTA	GGCCAATGAAACTGCTCAAGA
APOB	apolipoprotein B	Target	CCACCAAGATCAACTGCAAGGT	GGAGCAGTGGCTGGTCTTCA
ARNT	aryl hydrocarbon receptor nuclear translocator	Target	TCTAATGATAAGGAGCGGTTTGC	TATGATTTTCCCTGGCGAGTCT
B2M	beta-2-microglobulin	Endogenous	ACCTTCTGGTCCACACTGAGTTC	GGTCTCGATCCCACTTAACTATCTTG
MLXIPL	MLX interacting protein like	Target	CCCAAGTGGAAGAATTTCAAAGG	CTTCCTCCGCTCCACATACTG
CPT1A	carnitine palmitoyltransferase 1A	Target	CCTGAAGGTGCTGCTCTCCTA	CTCACCATCATCATCCAGATCTTG
CROT	carnitine O-octanoyltransferase	Target	GGGAAACGAAATTGGTTGGA	CGCAAAGTTGACATTCAGTTGTG
CYP2U1	cytochrome P450 family 2 subfamily U member 1	Target	AGAGAAAACAGTGCTCCAAGGGTAT	TGGCTGGGTCTCTGTGTACTGA
CYP7A1	cytochrome P450 family 7 subfamily A member 1	Target	TGACGCAGAGAAAGCCAAGTC	TTCAGAAGCTGCTTTCATTGCT
DGAT1	diacylglycerol O-acyltransferase 1	Target	CCTGAATTGGTGTGTGGTCATG	GATGCCGTACTTGATGAGGTTCTC
EGF	epidermal growth factor	Target	AACGGGAATGCCACTTGTGT	CCTTCCAAGTCAATCCTAAAGATACTG
ELOVL5	ELOVL fatty acid elongase 5	Target	CCTCTCGGCTGGCTGTACTT	CCTTCTTGTTGTAGGTCTGGATGTAG
ELOVL6	ELOVL fatty acid elongase 6	Target	AGCAGTTCAACGAGAACGAAGCC	TGCCGACCGCCAAAGATAAAG
ESRRA	estrogen related receptor alpha	Target	CAAGAGCATCCCAGGCTTCTC	CACCCAACACCAATACCTCCAT
FABP1	fatty acid binding protein 1	Target	AAGGTCGTCCAGAATGAGTTCAC	TTCCAACTGAACCACTGTCTTGA
FABP5	fatty acid binding protein 5 (psoriasis-associated)	Target	CCAATGGAGAATTGGTTCAACA	GTTCATGACGCATACCACCACTA
FADS1	fatty acid desaturase 1	Target	CCTTGTGAGGAAGTATATGAGCTCTCT	TCATCTGTCAGCTCTTTATTCTTAGTCG
FADS2	fatty acid desaturase 2	Target	TCCACCGCGACCTTGATTTA	TCGGTGATCTCAGAGTTCTTGGT
FADS3	fatty acid desaturase 3	Target	CCAGCACCTCTACTTCTTCCTGAT	CATGTATGCCAGATTTTCCACTTC
FOXA1	forkhead box A1	Target	ACAGCTACTACGCGGACACACA	ATGGTGCTCAGACCCGAGTT
HADH	hydroxyacyl-CoA dehydrogenase	Target	AGCTCTTCAAGAGGCTGGACAA	TTGTGATCTGCAAAGAGGAAGTG
HNF4A	hepatocyte nuclear factor 4 alpha	Target	GACCATGGGCAATGACACATC	GCACACAAGGCGCTGACA
HNF4G	hepatocyte nuclear factor 4 gamma	Target	GTCTTGGTGGAATGGGCTAAAT	CTCTCAACAGTGCCACCTGATC
HPRT1	hypoxanthine phosphoribosyltransferase 1	Endogenous	TCATTATGCCGAGGATTTGGA	CTCTTTCATCACATCTCGAGCAA
KLF10	Kruppel like factor 10	Target	ACGATCCCAGCATTTTGTTTG	TGGTGCCATCAGATTTGACACT
LIPC	lipase C, hepatic type	Target	CGTTACAGCAGTGTGGCTTCA	CCAGCAAGCCATCCATCAA
LPIN1	lipin 1	Target	CCGAGAGAAGGTGGTGGACAT	CTCTCCATTGTCTCCCAGTTTCA
MTTP	microsomal triglyceride transfer protein	Target	GAGGTCCAGGGTGGTCTAGCTATT	AGTTATTACCACGGTTACCCGATTT
NFKB1	nuclear factor of kappa light polypeptide gene enhancer in B-cells 1	Target	CCCACAGACGTTCATAGACAATTT	GAGGCTGGTTTTGTAATGTTGACA
NR1H3	nuclear receptor subfamily 1, group H, member 3	Target	CTGGGCATGATCGAGAAGCT	TGGGCCAAGGCGTGACT
NR2E1	nuclear receptor subfamily 2 group E member 1	Target	GGAATAGCACAATGGGCCATT	TTGTCACCATTCATGCCAGATA
PEX2	peroxisomal biogenesis factor 2	Target	CTCAGACTCCTAAGAAACCTTCAGAGA	ACTGATTCTGAGCACTCTGTTTGC
PLA2G12A	phospholipase A2, group XIIA	Target	CCCACTCTTTGGTGTTCATCTTAA	ATAGCACCTGTCGTGCTGGTT
PLCB2	phospholipase C beta 2	Target	AGATCTTCACTTCCTACCACTCCAA	GCGCTGTTTCTGGTTGATGA
POU2F1	POU class 2 homeobox 1	Target	CCCATACAGATCGCACAGGAT	GATGATAAACTGTGCTGGTTGCA
PPAP2A	phosphatidic acid phosphatase type 2A	Target	GGCCACTCTTCATTCTCCATGTAC	AGGCCCACGTAAATGGATACAG

PPARA	peroxisome proliferator activated receptor alpha	Target	GGCACTGAACATCGAATGTAGAATC	CCGAAAGAAGCCCTTGCAA
PPARD	peroxisome proliferator activated receptor delta	Target	GCATGTCTCACAACGCCATT	GCTGACTCCCCTCGTTTGC
PPARGC1A	peroxisome proliferator-activated receptor gamma, coactivator 1 alpha	Target	CTCTGGAACTGCAGGCCTAA	TGGAGAAGCCCTAAAAGGGTTAT
SCAP	SREBF chaperone	Target	AGATATCTCAGGCCTTCTACAACCA	AGTTTCAACAGTGGGTAGCAGC
SCD1	stearoyl-CoA desaturase (delta-9-desaturase)	Target	GGTGATGTTCCAGAGGAGGTACTAC	CAGCAATACCAGGGCACGAT
SREBF1	sterol regulatory element binding transcription factor 1	Target	CACGGAGGCGAAGCTGAATA	GCTTCTGGTTGCTCTGCTGAA
TBP	TATA box binding protein	Endogenous	CAGAATGATCAAACCGAGAATTGT	CTGCTCTGACTTTAGCACCTGTTAA
USF1	upstream transcription factor 1	Target	CCCTTATTCCCCGAAGTCAGA	GCGGCGTTCCACTTCATTAT
